# Synthesis, Morphology, and Hydrogen Absorption Properties of TiVMn and TiCrMn Nanoalloys with a FCC Structure

**DOI:** 10.1155/2018/5906473

**Published:** 2018-06-03

**Authors:** Bo Li, Jianding Li, Huaiyu Shao, Wei Li, Huaijun Lin

**Affiliations:** ^1^Institute of Applied Physics and Materials Engineering (IAPME), University of Macau, Macau; ^2^Institute of Advanced Wear & Corrosion Resistance and Functional Materials, Jinan University, Guangzhou 510632, China

## Abstract

TiVMn and TiCrMn alloys are promising hydrogen storage materials for onboard application due to their high hydrogen absorption content. However, the traditional synthesis method of melting and continuous necessary heat treatment and activation process are energy- and time-consuming. There is rarely any report on kinetics improvement and nanoprocessing in TiVMn- and TiCrMn-based alloys. Here, through ball milling with carbon black as additive, we synthesized face-centered cubic (FCC) structure TiVMn- and TiCrMn-based nanoalloys with mean particle sizes of around a few to tens of *μ*m and with the crystallite size just 10 to 13 nm. Differential scanning calorimetry (DSC) measurements under hydrogen atmosphere of the two obtained TiVMn and TiCrMn nanoalloys show much enhancement on the hydrogen absorption performance. The mechanism of the property improvement and the difference in the two samples were discussed from microstructure and morphology aspects. The study here demonstrates a new potential methodology for development of next-generation hydrogen absorption materials.

## 1. Introduction

For the realization of hydrogen energy society, it is crucial to develop low-cost and high-energy density hydrogen storage technologies and materials [1–5]. In the last decades, numerous types of hydrogen storage materials have been investigated for solid-state onboard storage of hydrogen [6, 7], for instance, metals and alloys [8–12], complex hydrides [13–16], chemical compounds [17–19], and carbon-based absorbents [20, 21]. However, none of these studied systems could entirely meet the technical requirements set by the US Department of Energy for onboard storage. Novel ideas and technologies are needed to develop future hydrogen storage materials.

Metal-/alloy-based hydrogen storage materials are thought to be promising candidates due to the good properties in capacity, kinetics and cycle ability, and so on, and these materials have presented excellent application performance in Ni-metal hydride (Ni-MH) rechargeable batteries [22–24], with low requirement on hydrogen storage capacity. However, preceding design and development in traditional interstitial metal-/alloy-based hydrogen storage materials suffer from limited capacity, poor kinetics, and lattice volume expansion. TiVMn- and TiCrMn-based alloys with bcc-Lave phase structure have been widely investigated for onboard hydrogen storage development due to the room temperature working temperature and possibly high hydrogen absorption capacity (3.5–4.2 wt%) [25–33]. However, synthesis of these alloys, normally by melting method, needs quite harsh conditions, which is also highly energy inefficient. Afterwards, the obtained alloys need strict conditions for heat treatment and hydrogen sorption activation processes before it may reversibly absorb and desorb hydrogen. Therefore, the kinetics of the TiVMn- and TiCrMn-based alloys should be enhanced. Ball milling technique is one of the most popular synthesis and downsizing methods to improve the sorption kinetics for hydrogen storage materials. However, we may hardly find any reports on synthesis of these two alloys by ball milling (or mechanical alloying) methods. The reason is that direct ball milling of the mixture of raw metals of Ti, V, and so on will result in sticking of the sample to the vessel wall and milling balls (see Figure S1). Here, we report that TiVMn and TiCrMn alloys with fine particle size and uniform nanostructured crystallite size were obtained through ball milling with carbon black as additive. Interestingly, these synthesized alloys are with a FCC structure, which is the first time to be reported in TiVMn- and TiCrMn-based alloys. Structure, morphology, and hydrogen storage properties of the TiVMn- and TiCrMn-based nanoalloys are discussed in this work.

## 2. Experimental Details

TiVMn and TiCrMn alloys were synthesized from Ti (−325 mesh, purity > 99.5%, Alfa Aesar),V (−325 mesh, purity > 99.5%, Alfa Aesar), and Mn (−325 mesh, purity > 99.5%, Alfa Aesar) metals and Ti (−325 mesh, purity > 99.5%, Alfa Aesar),Cr (−200 mesh, purity > 99%, Alfa Aesar) and Mn (−325 mesh, purity > 99.5%, Alfa Aesar) metals, respectively, by mechanical alloying method. Another 10 wt% carbon black (Sigma Aldrich) was added as additive during the milling process. The mechanical alloying process of these alloys was carried out with a rotation speed of 600 rpm and a milling duration of 10 h under Ar atmosphere using Fritsch P7 planetary micromiller. For a typical milling process, 0.5 g mixture of the raw metals with an atomic ratio of 1 : 1 : 1 and 0.05 g (10 wt%) carbon black was put into a 45 ml stainless steel vessel. Ten stainless steel milling balls with a diameter of 0.7 cm and an average weight of 1.5 g were used. The ball-to-sample ratio was 30 : 1.

The X-ray diffraction (XRD) measurements were carried out using a Rigaku diffractometer (Ultima IV) with CuK*α* radiation at a generator voltage of 40 kV and a current of 40 mA, to obtain the phase information of the samples. The analysis of the microstructure and elemental information was conducted using scanning electron microscope (SEM) (S3400N, Hitachi). It may investigate samples by both secondary electron (SE) and backscattered electron (BSE) signals. The SEM apparatus is attached with an energy-dispersive X-ray spectrometer (EDS). Differential scanning calorimetry (DSC) measurements under hydrogen atmosphere were carried out to study the hydrogen absorption properties of the alloys, through a Rigaku TP-8230 HP apparatus under a constant hydrogen pressure of 1 MPa with a flow rate of 200 ml/min.

## 3. Results and Discussion

**Figure 1:**
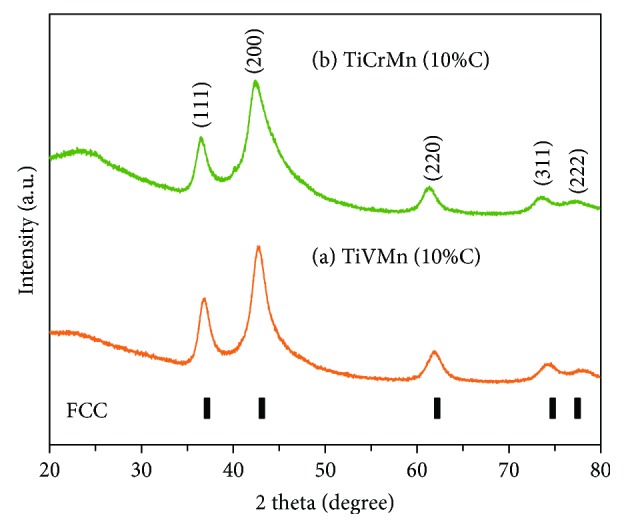
X-ray diffraction curves of (a) TiVMn-10%C and (b) TiCrMn-10%C nanoalloys with a FCC structure after 10 h mechanical alloying process.

From the XRD curves of the ball milled TiVMn-10%C and TiCrMn-10%C alloys which have been normalized to clearly present the phase compositions of each sample, we can see that both of these two reflection patterns are in perfect fitting with a FCC structure (face-centered cubic, space group: *Fm*-3 m no. 225), showing 5 reflections of (111), (200), (220), (311), and (222) at around 37°, 43°, 62°, 75°, and 78°, respectively. There is no obvious diffraction peak from the raw materials (Ti, V, Mn, and C or Ti, Cr, Mn, and C), which means the starting metal powders have been transformed into the new phase. The lattice parameter of the TiVMn alloy is *a* = 4.234 Å, and the one for the TiCrMn alloy is *a* = 4.270 Å. In Figure 1, we can see that the diffraction peaks present severe broadening, which indicates a rather fine nanocrystalline microstructure in these two samples. The average crystallite sizes of the TiVMn and TiCrMn nanoalloys were calculated to be 12.6 nm and 10.4 nm. As we discussed above, there is rarely any report on nanoprocessing synthesis of TiVMn- and TiCrMn-based hydrogen storage materials. Ball milling is the most popular synthesis technique applied in various hydrogen storage materials to obtain nanosize samples [34–46]. However, milling of the mixture of Ti-based materials such as Ti and V powders normally results in a melted metal state (see Figure S1) and not any powder sample can be obtained after the milling with a duration of 2 to 24 hours. Here, through addition of 10 wt% carbon black, we successfully synthesized nanostructured TiVMn and TiCrMn alloys with very fine nanocrystallite structures. The specific atomic position of Ti, V, Mn, and C in the TiVMn alloy with FCC structure (or Ti, Cr, Mn, and C in TiCrMn alloy), at this moment, is under study. Recently, we have made some progress and discussion in the Ti_50_V_50_-C alloy sample [47].

**Figure 2:**
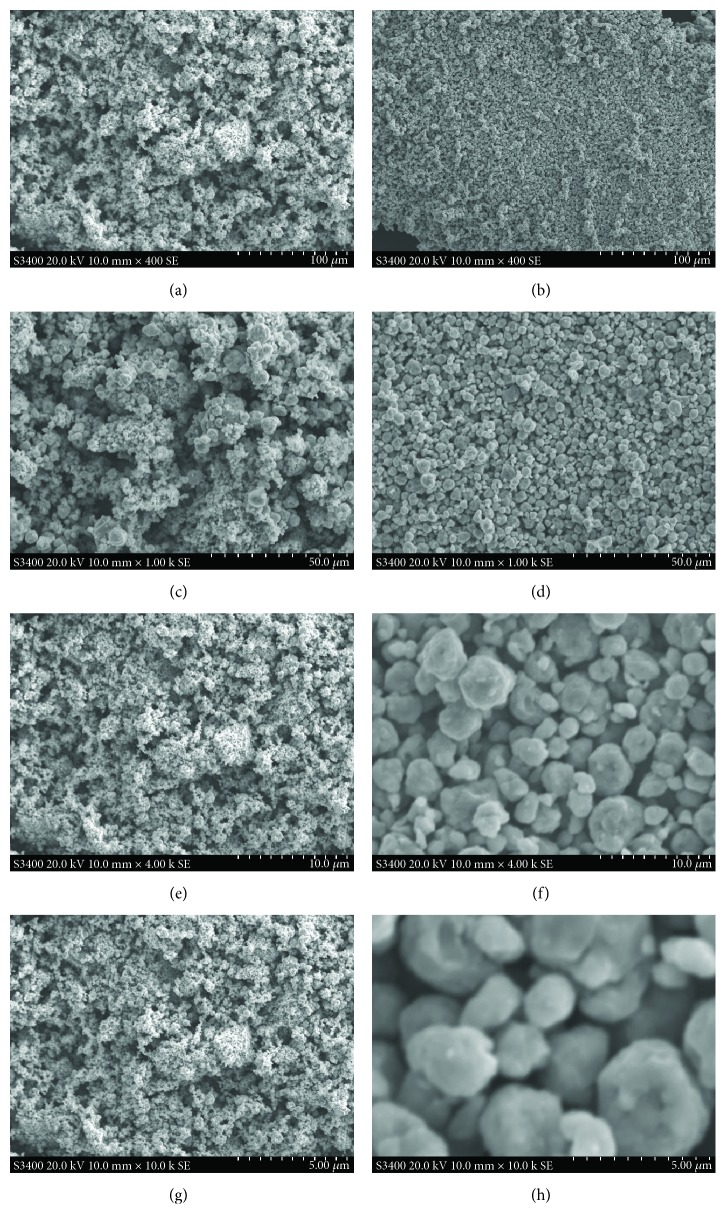
SE-SEM images of the synthesized TiVMn nanoalloy (a) 400x, (c) 1000x, (e) 4000x, and (g) 10,000x and TiCrMn nanoalloy (b) 400x, (d) 1000x, (f) 4000x, and (h) 10,000x after 10 h milling.

**Figure 3:**
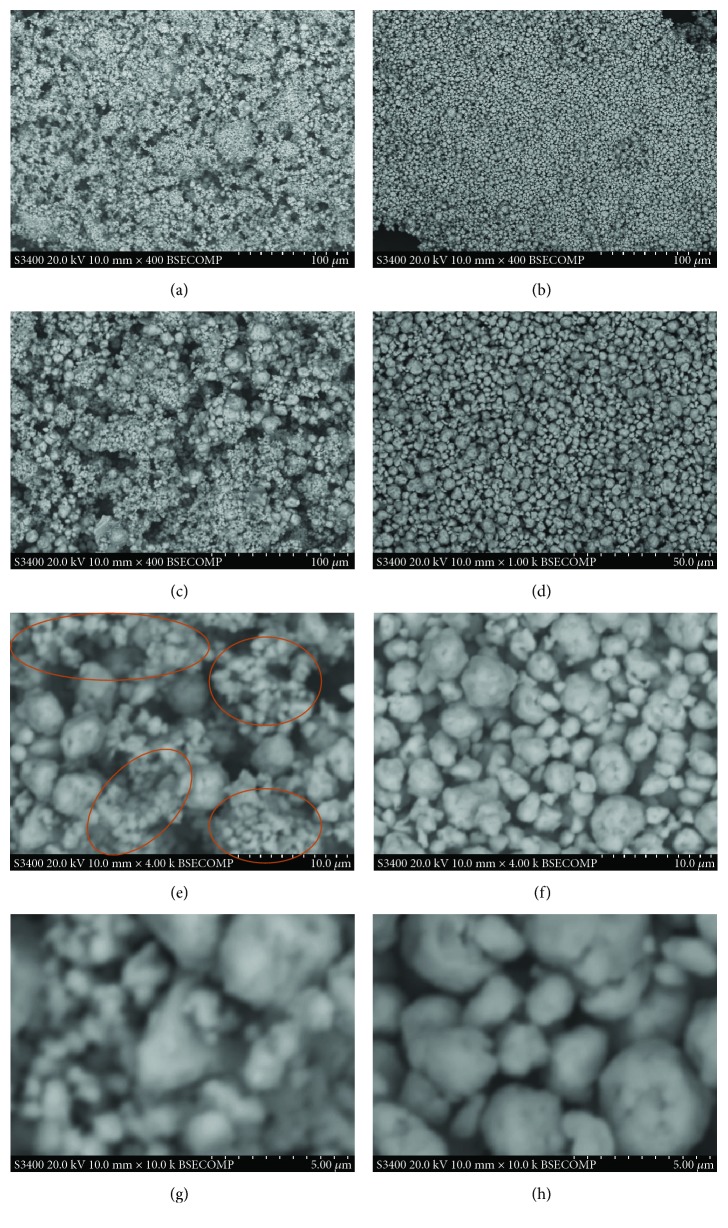
BSE-SEM images of the synthesized TiVMn nanoalloy (a) 400x, (c) 1000x, (e) 4000x, and (g) 10,000x and TiCrMn nanoalloy (b) 400x, (d) 1000x, (f) 4000x, and (h) 10,000x after 10 h milling.

Figures 2 and 3 are the SE-SEM and BSE-SEM images of the TiVMn and TiCrMn nanoalloy samples at different magnifications (400x to 10,000x). From these two figures, we may see the size and morphology of the two obtained samples after ball milling synthesis process. From Figures 2(a) and 2(b), we can see that both of the two samples show uniform morphology in a large range (ca. 300 *μ*m × 200 *μ*m). Figures 2(c) and 2(d) may be more clearly presenting some difference in the morphology of these two samples. The TiVMn alloy particles are with a larger size range than the TiCrMn ones. The particles of TiVMn in Figures 2(c) and 2(e) show a size from a few hundred nm to a few dozen *μ*m, while the ones for TiCrMn (Figures 2(d) and 2(f)) are in a size range mainly around 2 to 5 *μ*m. This morphology difference of the synthesized samples in the same milling conditions is thought to be attributed to the raw composition of starting metal materials. Ball milling method is a high-energy operation of repeated welding and fracturing of the raw mixture samples. In our former studies of Mg-Co-based materials, no additional composition is needed for the milling process [45]. However, in the milling process of TiVMn and TiCrMn from raw metal mixture, some additives, in this case carbon black, are essential to guarantee that powder samples can be obtained after 10 h milling. Figures 2(g) and 2(h) present some detailed morphology with high magnification for certain sample areas of the TiVMn and TiCrMn alloys, respectively. Ball milling method has been widely adopted in synthesis of hydrogen storage materials in nanostructure [48]. In our previous work, we have used ball milling technique to synthesize Mg-based metastable hydrogen storage alloys and we reported the Mg-Co-based metastable alloy with body-centered cubic structure which may absorb hydrogen at −15°C which is the lowest temperature reported so far for Mg-based material to absorb hydrogen [5, 45, 49, 50]. SEM as one scanning technique to obtain morphology of the samples may provide some key information to understand the formation mechanism of the obtained alloy from raw metal mixture. In our previous study [45], we comprehensively investigated the formation evolution process of Mg-Co nanoalloys milled from 0.5 to 400 h.

When we compare Figure 3 with Figure 2, the SE-SEM images may give us information about the three-dimensional morphology of the two alloy particle samples, while the BSE signal ones may be more sensitive in the composition difference of the particle surface. For the TiCrMn alloy in Figures 3(b), 3(d), 3(f), and 3(h), the BSE-SEM images indicate quite a uniform contrast, which demonstrates that the surface of the TiCrMn alloy is with quite a homogenous composition. For the TiVMn sample in Figures 3(a), 3(c), 3(e), and 3(g), the situation is a little different with that for the TiCrMn alloy sample. We can see that there are two domains with a little different contrast—the gray area and the white one. In Figure 3(e), we can also clearly observe some areas with much smaller particle size compared with the nearby ones (in orange circles). From XRD analysis, there is only one FCC phase in both of these two samples. However, the SE-SEM and BSE-SEM images can tell some obvious difference in particle size and surface composition.

**Figure 4:**
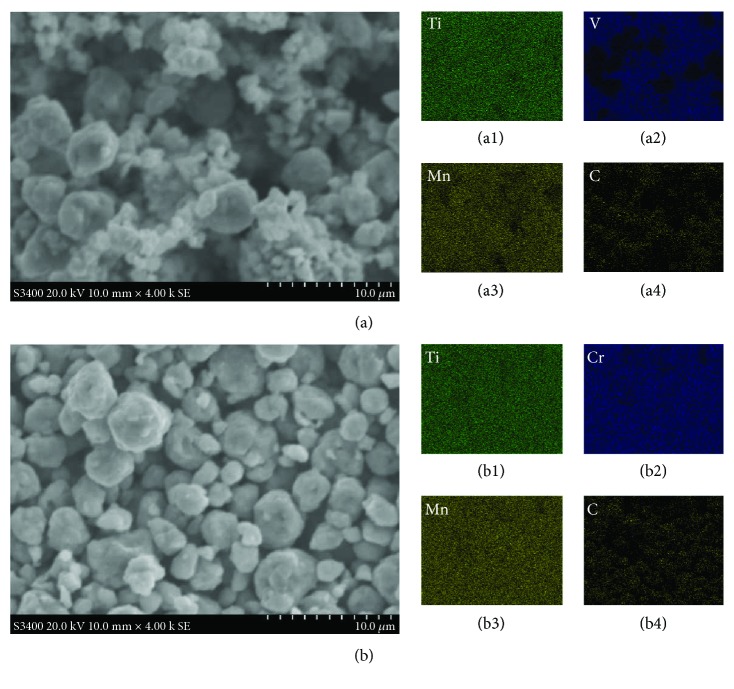
SE-SEM images of the (a) TiVMn and (b) TiCrMn nanoalloys. EDS mappings of (a1) Ti, (a2) V, (a3) Mn, and (a4) C for the corresponding TiVMn area in (a) and (b1) Ti, (b2) Cr, (b3) Mn, and (b4) C for the corresponding TiCrMn area in (b).

Figure 4 presents the elemental analysis results of the particles in the TiVMn and TiCrMn alloy samples. From Figure 4(b1)–(b4), we may see that after 10 h ball milling, Ti, Cr, and Mn are homogenously distributed throughout the TiCrMn sample, which demonstrates that a uniform compound is formed after the milling process. Carbon black is introduced as additive for milling assistance of TiVMn and TiCrMn samples so that it is possible to obtain powder samples after the milling process, while not the entire sample sticks to the inner wall of the milling vessel or surface of the balls. After the milling, C element is also well dispersed in the sample. Since we cannot detect any C reflection peaks or amorphous background from XRD or find obvious isolate C areas by EDS mapping of the sample, we may conclude that C is in the composition of the FCC structure compound. From the EDS mapping of the TiVMn sample, again, we found some difference with the TiCrMn one. Ti and Mn are homogenously dispersed in the sample, but different with Cr in the TiCrMn sample, V in the TiVMn sample is not that uniformly distributed. From Figure 4(a) and Figure 4(a2), we can see that the smaller particles (less than 10 *μ*m) in the TiVMn sample is much more V rich than the larger ones. We believe that this difference may contribute to different hydrogen storage properties of these two samples. It should be pointed out that it is a universal factor that synthesis by ball milling will introduce some contamination from milling vessel/balls to the sample. In our cases, we found that some Fe element from the milling tools is around 3–8 wt%, which was attributed to the long-time milling process between samples and stainless steel vessels and milling balls.

**Figure 5:**
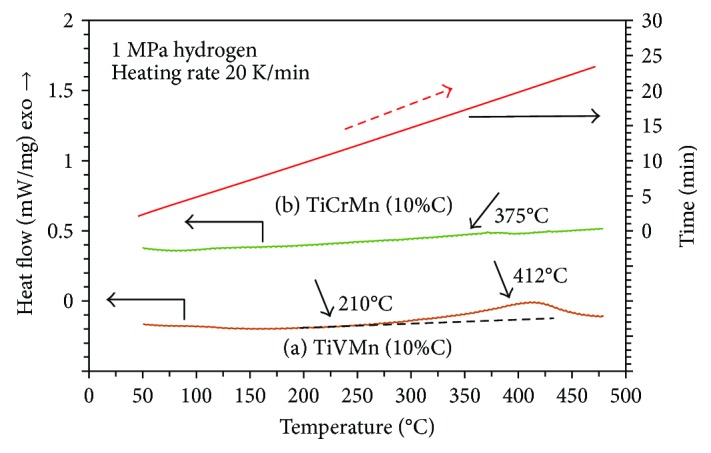
High pressure DSC curves of the synthesized TiVMn and TiCrMn nanoalloy samples in 1 MPa hydrogen atmosphere. Red solid line presents the time-heating program information.

Figure 5 presents the high-pressure DSC curves of the synthesized TiVMn and TiCrMn alloy samples with FCC structure, under a hydrogen atmosphere of 1 MPa. Hydrogen pressure DSC is a very simple and essential technique to test the hydrogen absorption and desorption properties of samples. A quite small amount sample is enough (around 10 mg) to obtain some temperature and hydrogen pressure information necessary for the absorption and desorption reactions. The temperature program in Figure 5 was set as from room temperature to 500°C with a heating rate of 20 K/min (red solid line). In Figure 5, we can see that the TiVMn and TiCrMn nanoalloys may show hydrogen absorption (exothermic reaction peaks) before 450°C without any activation process. According to our experiences in study of hydrogen storage alloys for DSC measurements under hydrogen atmosphere, especially for metastable alloys without any phase transfer process in the measurement temperature range, exothermic peaks are attributed to hydrogen absorption reactions [44–46, 51, 52]. The hydrogenation peak temperatures under 1 MPa hydrogen are 412 and 375°C for the TiVMn and TiCrMn nanoalloys, respectively. It is worth noting that although the DSC measurement of samples heated under hydrogen pressure at certain time and temperature point may be similar with the conditions of taking hydrogen absorption measurements, it is still quite different. The reason is that the temperature of the sample at DSC measurement is under heating and the real temperature of the sample is increasing at a very high rate (20 K/min in this case). As a result, the peak temperature derived from the DSC measurements under certain hydrogen pressure actually is much higher than the one needed for hydrogen absorption measurements at the same constant temperature. This means these two alloys may absorb hydrogen at a much lower temperature than 412°C for TiVMn nanoalloy and 375°C for the TiCrMn one without heat treatment and activation. When we compare the hydrogen absorption reaction peaks of these two samples, we can see that although TiCrMn nanoalloy may absorb hydrogen with a peak temperature at around 37°C lower than the TiVMn one, the absorption reaction peak of TiVMn alloy is much larger than the one from TiCrMn alloy. This means a much larger ratio of the TiVMn alloy may start to absorb hydrogen than the TiCrMn one at the corresponding peak temperature. Another essential point is that the TiVMn sample may start to absorb hydrogen at around 210°C from the DSC measurement. This means that in hydrogen absorption under constant temperature, TiVMn nanoalloy may start the absorption process at a temperature much lower than 210°C. The difference in the hydrogen absorption properties of these two nanoalloy samples is thought to be the V-rich area and partially smaller particle size in the TiVMn nanoalloy sample compared to the TiCrMn one, observed from the SEM characterization techniques.

The TiVMn and TiCrMn alloys synthesized by melting method have been widely investigated as hydrogen storage materials [28, 31, 53–58]. The two alloys after melting are usually with bcc-C14 Lave phase structures, and they may start to absorb hydrogen after a strict heat treatment at a temperature greater than 600°C and an activation process at high temperature and high pressure hydrogen atmosphere, which is quite time- and energy-consuming. So, we may conclude that via ball milling with carbon black, the obtained TiVMn and TiCrMn nanoalloy samples with a FCC structure show much enhanced hydrogen absorption performance than the TiVMn- and TiCrMn-based alloys obtained by melting method, implying a novel development methodology of future hydrogen storage materials.

## 4. Conclusions

TiVMn and TiCrMn nanoalloys with a FCC structure (face-centered cubic, space group: *Fm*-3 m no. 225) were synthesized by ball milling with carbon black as additive. The lattice parameter and crystallite size of the TiVMn nanoalloy are *a* = 4.234 Å and 12.6 nm, respectively. The ones for the TiCrMn alloy are *a* = 4.270 Å and 10.4 nm. The SE- and BSE-SEM observations at different magnifications show that the TiVMn alloy particles (a few hundred nm to a few dozen *μ*m) are with a larger size range than the TiCrMn ones (mainly 2 to 5 *μ*m). In the TiVMn nanoalloy, there are domains with smaller particle size and V-rich composition compared with the other area. The morphology and microstructure differences contribute to the different hydrogen absorption properties by DSC measurements under hydrogen pressure. The TiVMn nanoalloy and the TiCrMn one show absorption peaks at 412 and 375°C, respectively. But the absorption reaction is much stronger and starts at much lower temperature (210°C) in the TiVMn nanoalloy than that in the TiCrMn one. This work implies a new development methodology of future hydrogen storage materials.

## Data Availability

The data used to support the findings of this study are available from the corresponding author upon request.

## Supplementary Materials

Supplementary Materials Figure S1: direct ball milling of the mixture of raw metals of Ti, V, and so on will result in sticking of the sample to the vessel wall and milling balls. No powders can be collected after ball milling for 2 to 24 hours. Figure S2: dark-field TEM images of TiVMn (a) and TiCrMn (b).
