# Corrigendum to “Synthesis, Morphology, and Hydrogen Absorption Properties of TiVMn and TiCrMn Nanoalloys with a FCC Structure”

**DOI:** 10.1155/2019/8567174

**Published:** 2019-07-31

**Authors:** Bo Li, Jianding Li, Huaiyu Shao, Wei Li, Huaijun Lin

**Affiliations:** ^1^Institute of Applied Physics and Materials Engineering (IAPME), University of Macau, Macau SAR, China; ^2^International Institute for Carbon-Neutral Energy Research (WPI-I2CNER), Kyushu University, Fukuoka 819-0395, Japan; ^3^Institute of Advanced Wear & Corrosion Resistance and Functional Materials, Jinan University, Guangzhou 510632, China

In the article titled “Synthesis, Morphology, and Hydrogen Absorption Properties of TiVMn and TiCrMn Nanoalloys with a FCC Structure,” [1], there was a former affiliation missing for the third author, which is “International Institute for Carbon-Neutral Energy Research (WPI- I2CNER), Kyushu University, Fukuoka 819-0395, Japan”. The corrected authors' list and affiliations are shown above.

## References

[1] Li B., Li J., Shao H., Li W., Lin H. (2018). Synthesis, morphology, and hydrogen absorption properties of TiVMn and TiCrMn nanoalloys with a FCC structure. *Scanning*.

