# Supplementary material for: Synthesis, Morphology, and Hydrogen Absorption Properties of TiVMn and TiCrMn Nanoalloys with a FCC Structure
Source: Scanning. 2018 Jun 3;2018:5906473. doi: 10.1155/2018/5906473 (PMC6008909; doi:10.1155/2018/5906473)
Supplement: Supplementary Materials — Figure S1: direct ball milling of the mixture of raw metals of Ti, V, and so on will result in sticking of the sample to the vessel wall and milling balls. No powders can be collected after ball milling for 2 to 24 hours. Figure S2: dark-field TEM images of TiVMn (a) and TiCrMn (b). [file 5906473.f1.pdf]

Research Article

Synthesis, Morphology and Hydrogen Absorption Properties of TiVMn and TiCrMn Nano Alloys with a FCC Structure

Bo Li[1], Jianding Li[1], Huaiyu Shao[1*], Wei Li[2] and Huaijun Lin[2*]

[1]Institute of Applied Physics and Materials Engineering (IAPME), University of Macau, Macau SAR, China

[2]Institute of Advanced Wear & Corrosion Resistance and Functional Materials, Jinan University, Guangzhou 510632, China

[*]Corresponding author: hshao@umac.mo (H. Shao), hjlin@jnu.edu.cn (H. Lin)

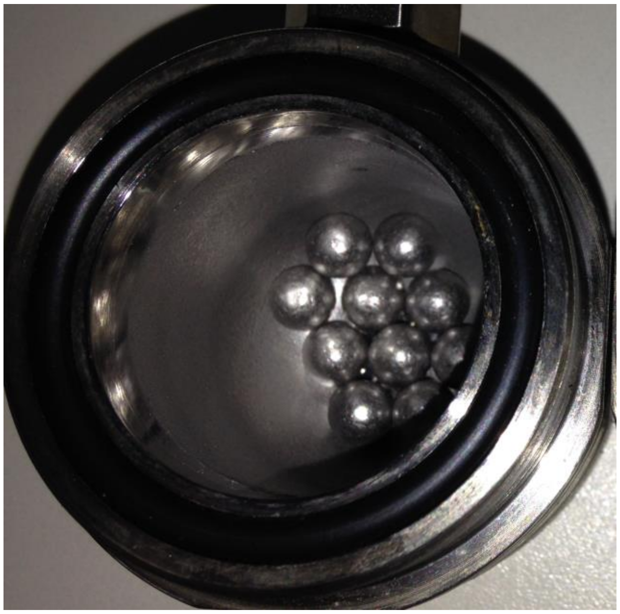

Figure S1. Direct ball milling of the mixture of raw metals of Ti, V, etc. will result in sticking of the sample to the vessel wall and milling balls. No powders can be collected after ball milling for 2 to 24 hours.

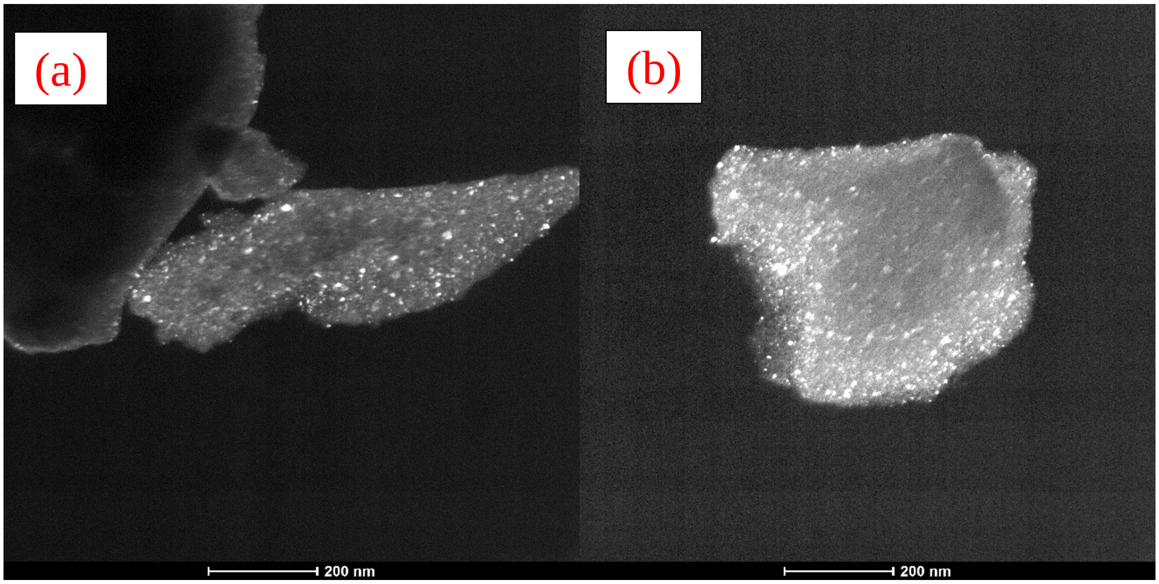

Figure S2. Dark-field TEM images of TiVMn (a) and TiCrMn (b).
